# Curcumin supplementation increases longevity and antioxidant capacity in *Caenorhabditis elegans*

**DOI:** 10.3389/fphar.2023.1195490

**Published:** 2023-06-06

**Authors:** Jianing Xu, Pengyun Du, Xiaoyu Liu, Xiao Xu, Yuting Ge, Chenggang Zhang

**Affiliations:** School of Life Sciences, Beijing University of Chinese Medicine, Beijing, China

**Keywords:** curcumin, *Caenorhabditis elegans*, ROS, RNA-seq, MAPK pathway

## Abstract

Curcumin is well known as a potent antioxidant and free radical scavenger and has great potential for anti-aging applications. In this study, we investigate the molecular mechanism of curcumin in prolonging the lifespan of *C. elegans*. Four concentrations of curcumin (10, 25, 50, and 100 µM) were administered, and the optimal treatment concentration was determined by analyzing the nematode lifespan, physiology, and biochemistry. Additionally, RNA-seq and qRT-PCR were performed to explore the antioxidant effect of curcumin and its underlying mechanism. Results revealed that curcumin could significantly improve the survival capacity of *C. elegans* without influencing its growth. Curcumin was observed to significantly decrease the levels of reactive oxygen species (ROS) under extreme conditions such as heat stress and paraquat stress. In addition, curcumin increased the amount of nematode mitochondrial DNA (mtDNA) replication. RNA-seq results revealed that the underlying mechanism of curcumin in *C. elegans* is related to the mitogen-activated protein kinase (MAPK) pathway. qRT-PCR results confirmed that the expression of oxidative stress-related genes (sod-1, sod-2, sod-3, gst-4) was increased, and the expression of MAPK signaling pathway-related genes (sek-1, pmk-1, nsy-1) was significantly downregulated. Furthermore, the administration of curcumin extended the lifespan of nematodes, potentially through the enhancement of oxidative stress resistance and the downregulation of the MAPK signaling pathway. These findings improve our understanding of both lifespan extension and the potential mechanism of curcumin in *C. elegans.*

## 1 Introduction

Aging and age-related disorders have emerged as significant medical and social concerns. Extensive research has indicated that the aging process is influenced by a complex interplay of various factors, including genetic and epigenetic interactions (Campisi et al., 2019; López-Otín et al., 2023). Aging is characterized by a decrease in various physiological activities of the organism and is accompanied by changes in the internal and external environment of the organism. Senescence is an irreversible inhibition of growth, a defect in tissue renewal and function, and the cessation of cellular replication, limiting the proliferative life span of cells (Tosato et al., 2007; Childs et al., 2015). Current scientific investigations into the fundamental molecular mechanisms and key pathways of aging have primarily concentrated on a select set of highly conserved biochemical mechanisms and pathways. These mechanisms and pathways play crucial roles not only in aging but also in the maintenance of cellular and organismal homeostasis (Flatt and Partridge, 2018; Kulminski et al., 2019). Because of their short and reproducible lifespan (18 days at 20°C), *Caenorhabditis elegans* is an ideal model organism to study the aging process. The entire genome of *C. elegans* has been sequenced, and most of the signaling pathways are evolutionarily consistent, hence attracting attention in lifespan-related research. *C. elegans* may provide an effective tool for screening genes and compounds that prolong the human lifespan (Palikaras, Lionaki, and Tavernarakis, 2015).

Anti-aging has become a worldwide scientific concern and although aging is inevitable, the process of aging can be regulated to a large extent. The theory of oxidative stress stands out as one of the prominent explanations for the underlying mechanisms of aging (Łuczaj, Gęgotek, and Skrzydlewska, 2017). Excessive production of ROS in the body or insufficient antioxidant defenses can lead to oxidative damage in the body, known as oxidative stress. Redox homeostasis is essential for the survival of organisms and is maintained by antioxidant enzymes, substrate supply, and self-repair. Moreover, ROS are a byproduct of oxygen metabolism and ATP production. Various factors such as radiation, smoking, alcohol abuse, drugs, and environmental pollution can induce oxidative stress in the body and produce ROS clusters far beyond their own antioxidant function, leading to the development of many aging-related diseases (Brieger et al., 2012). During the inflammatory response, neutrophil activation leads to increased production of ROS, an important intrinsic immune molecule that regulates the intrinsic immune response both through activation of the MAPK pathway and through multiple pathways (Fan et al., 2020). The body’s ability to scavenge oxidative stressors such as reactive oxygen species can be improved by a variety of means, including physiological signals, dietary components, and drugs. Many natural bioactive products and plant extracts have antioxidant life-extending abilities, including caffeic acid (Li et al., 2021), ginsenosides (Wijaya et al., 2022), blueberry extract (Albrahim and Alonazi, 2022), and chayote (Gavia-García et al., 2023).

Curcumin is a rare natural pigment containing diketones isolated from the rhizomes of the Curcuma longa (Nelson et al., 2017). Since its first discovery in 1815, curcumin has become one of the most broadly used natural pigments, with non-toxic and harmless features. It is a food additive widely used in food production and approved by the JECFA (The Joint FAO/WHO Expert Committee on Food Additives) (Priyadarsini, 2014). The ADI (adequate daily intake) value of curcumin is 0–3 mg/kg (Kocaadam and Şanlier, 2017). Curcumin is an important polyphenol in food, which reduces oxidative stress and regulates various body functions. Moreover, curcumin can significantly reduce intracellular lipid peroxidation and improve the antioxidant effect of the body to prolong life (Lee et al., 2010; Shehzad et al., 2017). It enhances the cell’s capacity to resist protein carbocation and regulates lipid peroxidation and mitochondrial permeability transition by increasing antioxidant enzyme activity (Kocaadam and Şanlier, 2017). Curcumin is an important biochemical antioxidant that modulates cellular immune responses and has anti-inflammatory effects, thereby reducing the damage caused by inflammation but improving cardiovascular function (El-Saadony et al., 2022). As an effective antioxidant, curcumin has great potential in reducing cellular damage, specifically mitochondrial damage, caused by ROS production. The use of antioxidant active agents to reduce free radicals in the body and protect mitochondria is an important strategy to slow down human aging (Yang et al., 2020). However, our current understanding of the mechanisms underlying the association between curcumin, mitochondrial protection, and lifespan extension remains limited.

With the extensive use of novel technologies and methods in the pharmaceutical field, high-throughput sequencing technology can reflect the gene expression status and reveal the mechanism of action of drugs (Tourasse, Millet, and Dupuy, 2017; Schumacker et al., 2019; Yu et al., 2021). These techniques can be used to further study the pharmacology, pharmacokinetics, and mechanism of action of curcumin. Herein, four concentrations of curcumin (10, 25, 50, and 100 µM) were administered to *C. elegans,* and the optimal treatment concentration was determined by analyzing the lifespan, physiological, and biochemical indices of the nematodes. Additionally, RNA-seq and qRT-PCR techniques were used to explore the mechanism of curcumin against oxidative stress and its lifespan extension.

## 2 Materials and methods

### 2.1 *C. elegans* strains and handling conditions

The wild-type strain N2 used in this study was obtained from *Caenorhabditis* Genetics Center (CGC, University of Minnesota, MN, United States). All strains were cultivated on nematode growth medium (NGM) plates seeded with live *E. coli* OP50 as food and incubated at 20°C (unless otherwise stated). For all worms, age-synchronized eggs were obtained using bleach (0.5 M NaOH, 5% NaClO) to separate embryos from the pregnant hermaphrodites. OP50 was grown in Luria-Bertani (LB) broth for 24 h in an incubator at 37°C. Curcumin (CAS: 458-37-7, Macklin, Shanghai, China; shelf time: 2 years) was dissolved in dimethyl sulfoxide (DMSO). A final DMSO concentration of 0.1% (v/v) was maintained under all conditions.

### 2.2 Lifespan measurement

Notably, life measurement require special NGM plates (Sutphin and Kaeberlein, 2009) containing 50 µM 5-fluoro-20-deoxyuridine (FUdR, Macklin, Shanghai, China) to prevent their progeny from hatching. N2 nematodes synchronized at the L4 stage were transferred to the NGM plates [treated with or without curcumin (10, 25, 50, 100 µM)]. Survival analysis was conducted to assess the daily survival of nematodes from the egg stage until the death of all individuals at a temperature of 20°C. Each experimental group consisted of approximately 30 nematodes. Lifespan comparisons were performed using GraphPad 8 software (GraphPad Software Inc., San Diego, CA, United States). The experiments were repeated at least three times to ensure reliability and reproducibility of the results.

### 2.3 Exercise indicator measurement

N2 nematodes synchronized at the L4 stage were transferred to fresh NGM plates [treated with or without curcumin (10, 25, 50, 100 µM)] and cultured for 72 h.

Head swing frequency was quantified as the number of head swings observed within a 1-min interval. Nematodes were placed in M9 buffer on a glass slide and observed under a microscope. Each complete back-and-forth motion of the head was considered as one swing. Approximately 30 nematodes were analyzed for each treatment group.

Body bending frequency was determined by recording the number of body bends observed within a 1-min timeframe. A single bend was defined as the movement of the nematode over one wavelength in relation to the long axis of its body. Thirty nematodes were assessed in each experimental group.

### 2.4 Reproduction assay

After synchronization, worms were transferred to different plates as described in Section 2.3. The adult nematodes were transferred to freshly prepared NGM plates daily until the end of the reproductive period. The hatched offspring were counted after 2 days. The eggs of 10 nematodes were counted in each group.

### 2.5 Determination of the effect of curcumin on the proliferation of *E. coli* OP50

Four holes were punched using a gun head (Lablead, Beijing, China) (10–100 µL) on the LB agar. The agar was removed from the hole using a sterilized needle. Then streptomycin, fungicidin, water, and Curcumin (10, 25, 50, and 100 µM) were injected into the hole and allowed to dry before evenly coating *E. coli* OP50 on the LB medium. The size of the inhibition zone was measured after 3 days of incubation. Experiments were repeated three times.

### 2.6 Stress resistance experiments

After synchronization, L4 nematodes were transferred to a medium containing different concentrations of curcumin and cultured for 48 h. Following the experimental treatments, nematodes were collected using M9 buffer and subjected to three consecutive rinses to remove any adhered *E. coli* OP50 from their body surface. Subsequently, the nematodes were transferred to NGM medium devoid of *E. coli* OP50. Upon completion of external stimulation under various conditions, the nematodes were returned to NGM/OP50 and cultured at 20°C for a duration of 12 h. The experimental procedures were repeated at least three times to ensure robustness and reliability of the results.

#### 2.6.1 Thermorecovery assay

The nematodes were maintained and treated as described above. *C. elegans* survival was measured after 4 h at 35°C followed by a recovery period of 12 h at 20°C. The worms unresponsive to any mechanical stimuli were considered dead. Each plate contained 30 nematodes, and each experiment was performed in triplicate.

The level of ROS was measured using the ROS kit (20,70-dichlorofluorescein diacetate (H2DCFDA) (Jiancheng biotechnology, China, Ex/Em 488/525 nm). Subsequently, the worms were maintained and treated as described above. After being exposed to curcumin for 3 days, the nematodes were placed in a 35°C incubator for 4 h before incubation with 100 µM H2DCFDA for 30 min. The fluorescence content of each group of nematodes was measured using a Type InfiniteF200Pro microplate reader (Tecan, Männedorf, Switzerland; Ex/Em 488 nm/525 nm) to measure the absorbance. Three independent experiments were performed, each with 30 nematodes.

#### 2.6.2 Fluorescent imaging of oxidative stress

N2 worms were maintained and treated as described above. The nematodes were soaked in 50 µM paraquat solution for 6 h. Next, collected and washed twice with M9 buffer. Subsequently, incubated with 100 µM H2DCFDA in the dark for 30 min. Nematodes were washed with M9 buffer and anesthetized [10 µM levamisole (Macklin, Shanghai, China)] before placing them on a 2% agarose pad. ROS production was analyzed by imaging under a fluorescence microscope (Olympus X71, Tokyo, Japan). The levels of reactive oxygen species (ROS) were quantified in a minimum of 15 nematodes. Imaging was conducted using 470 nm excitation, and the emitted green fluorescence was recorded at 550 nm. ImageJ software was employed to analyze the fluorescence intensity, with background signals subtracted for accurate quantification. The assay was repeated three times to ensure reproducibility and validity of the results.

### 2.7 Effect of total antioxidant capacity (T-AOC) in *C. elegans*

After synchronization, L4 *C. elegans* were incubated on different plates for 72 h, the *C. elegans* were rinsed three times, centrifuged, 1,000 nematodes were diluted to 1 mL with M9 buffer, the supernatant was homogenized by sonication and the nematode homogenate protein concentration was determined by the BCA method. Total anti-oxidation capacity (T-AOC) levels were measured using commercial kits following the manufacturer’s instructions (A015e1, Jiancheng biotechnology research institute, Nanjing, China), respectively. The absorbance was measured at 520 nm using a microplate reader. Each sample was examined three times.

### 2.8 Measurement of mitochondrial copy number

To perform rapid relative quantification of mitochondrial DNA (mtDNA) copy number, the nuclear DNA (nDNA) was used as an internal reference. The sequences of nDNA and mtDNA of the N2 strain of *C. elegans* were obtained from the GenBank database (NC_001328.1). The multiplex PCR primer design software MPprimer (Shen et al., 2010) and primer specificity evaluation software MFE primer V3. 0 (https://mfeprimer3.igenetech.com/spec) (Qu et al., 2012) were used to design primers for the PCR amplification of nematode nDNA and mtDNA. The primers were synthesized by Shanghai ShengGong Inc. Real-time PCR was performed on total nematode DNA (approximately 1,000 nematodes per group) using BBI’s ONE-4-ALL Genomic DNA Mini-Preps Kit (Order No. B618503) and SYBR^®^ Premix Ex kit Taq™ kit following the manufacturer’s instructions. The data was processed three times in parallel per sample according to the ΔΔCT threshold cycle method, and the average of the final 3 times obtained the Ct value of the sample. The specific primers included:

mtDNA: forward, 5′-TCG​TCT​AGG​GCC​CAC​CAA​GGT​T-3′

reverse, 5′-CTT​CTA​GCA​CGG​ATG​GCC​CCA​A-3′

nDNA: forward, 5′-CGG​CGA​TGT​GAA​TGC​GTC​CGA​T-3′

reverse, 5′-ATC​CCC​AGT​TCA​ATC​CTC​CGG​CA-3′

### 2.9 RNA-seq

Synchronized nematodes at the L4 stage were cultivated, and a total of 12,000 nematodes were allocated into two groups. The experimental group received a treatment of 25 µM curcumin, while the control group was maintained under normal culture conditions. After 72 h of administration, repeated washes were performed to remove *E. coli* OP50 from the *C. elegans* body surface and any residual drug was removed by liquid nitrogen freezing. Samples were then analyzed through Biomarker Technologies (Beijing, China). A total of 1 µg RNA per sample was used as input material for the RNA sample preparations. Sequencing libraries were generated using the NEB Next Ultra TM RNA Library Prep Kit for Illumina (NEB, Ipswich, MA, United States) following the manufacturer’s recommendations. Index codes were added to attribute sequences to each sample. The specified genome was used as a reference for the analysis (https://ftp.ncbi.nlm.nih.gov/genomes/all/GCF/000/002/985/GCF_000002985.6_WBcel235/(WBcel235) accessed on 18 October 2022). Differential expression analysis of three samples was performed using edgeR. A FDR <0.01 and Fold Change ≥2 were set as the threshold for significantly differential expression. Relevant sequencing results have been uploaded to the NCBI database (SUB12998438, PRJNA949877).

### 2.10 Quantitative real-time polymerase chain reaction (qRT-PCR)

Total nematode RNA was extracted using the TaKaRa Mini BEST Universal RNA Extraction Kit (approximately 2,000 per group). RNA was reverse transcribed to cDNA using the TaKaRa Prime Script RT Master Mix Kit according to the manufacturer’s protocol. The resulting cDNA was then subjected to real-time PCR using the SYBR^®^ Premix Ex Taq™ kit, following the manufacturer’s instructions. The data were calculated using ΔΔCT threshold cycling method, with the act-1 gene acting as the internal reference. The primers were designed and synthesized by Shanghai ShengGong Inc. according to the standard. The primer sequences are shown in Table 1.

**TABLE 1:** Gene-specific primers used in RT-PCR.

Genes	Primers
act-1	forward, 5′-TCG​GTA​TGG​GAC​AGA​AGG​AC-3′
	reverse, 5′- CAT​CCC​AGT​TGG​TGA​CGA​TA-3′
daf-2	forward, 5′-GTT​GAT​AAT​GCT​GCC​GAG-3′
	reverse, 5′- ATC​CCG​GTC​CGA​TTT​CTT-3′
daf-16	forward, 5′-ATC​GTG​TGC​TCA​GAA​TCC-3′
	reverse, 5′- ATG​AAT​AGC​TGC​CCT​CC-3′
sod-1	forward, 5′-GAG​TCG​GAG​ACA​AGG​CAG​AAG​AG-3′
	reverse, 5′- AGC​AGC​GAG​AGC​AAT​GAC​ACC-3′
sod-2	forward, 5′-TCG​CTG​TTC​AAG​GAT​CAG​GAT​GG-3′
	reverse, 5′- CAA​GTC​CAG​TTG​TTG​CCT​CAA​GTG-3′
sod-3	forward, 5′-CGA​GCT​CGA​ACC​TGT​AAT​CAG​CCA​TG-3′
	reverse, 5′- GGG​GTA​CCG​CTG​ATA​TTC​TTC​CAG​TTG-3′
gst-4	forward, 5′-TCC​GTC​AAT​TCA​CTT​CTT​CCG-3′
	reverse, 5′- CAC​CAT​GAG​TCC​AAT​GAT​TGC​A-3′
pmk-1	forward, 5′-TGC​TGA​CGA​AGA​GGA​AGA​TGA​GG-3′
	reverse, 5′- AAC​TGC​CTT​GGG​ATT​GTA​AGA​TGT​C-3′
sek-1	forward, 5′-CGA​GTC​CGA​AGA​GAT​TGA​GAT​TGC-3′
	reverse, 5′- CCC​GCT​CTG​CCT​GTG​CTG-3′
nsy-1	forward, 5′-GTA​TTG​GTG​TTG​GCG​GAG​GTT​C-3′
	reverse, 5′- ACT​GGC​TGA​CGG​CGT​TGA​C-3′

### 2.11 Statistical analysis

All experiments were performed in triplicate. The *p*-values were calculated using the log-rank test. The numerical data were analyzed using a Student’s t*-*test. The values are shown as the mean ± SEM. Significant statistical differences were set significant at *p* < 0.05 (*p < 0.05, **p < 0.01, and ***p < 0.001).

## 3 Results

### 3.1 Effect of curcumin on physiological indicators of *C. elegans*

#### 3.1.1 Curcumin prolongs *C. elegans* lifespan

**FIGURE 1:**
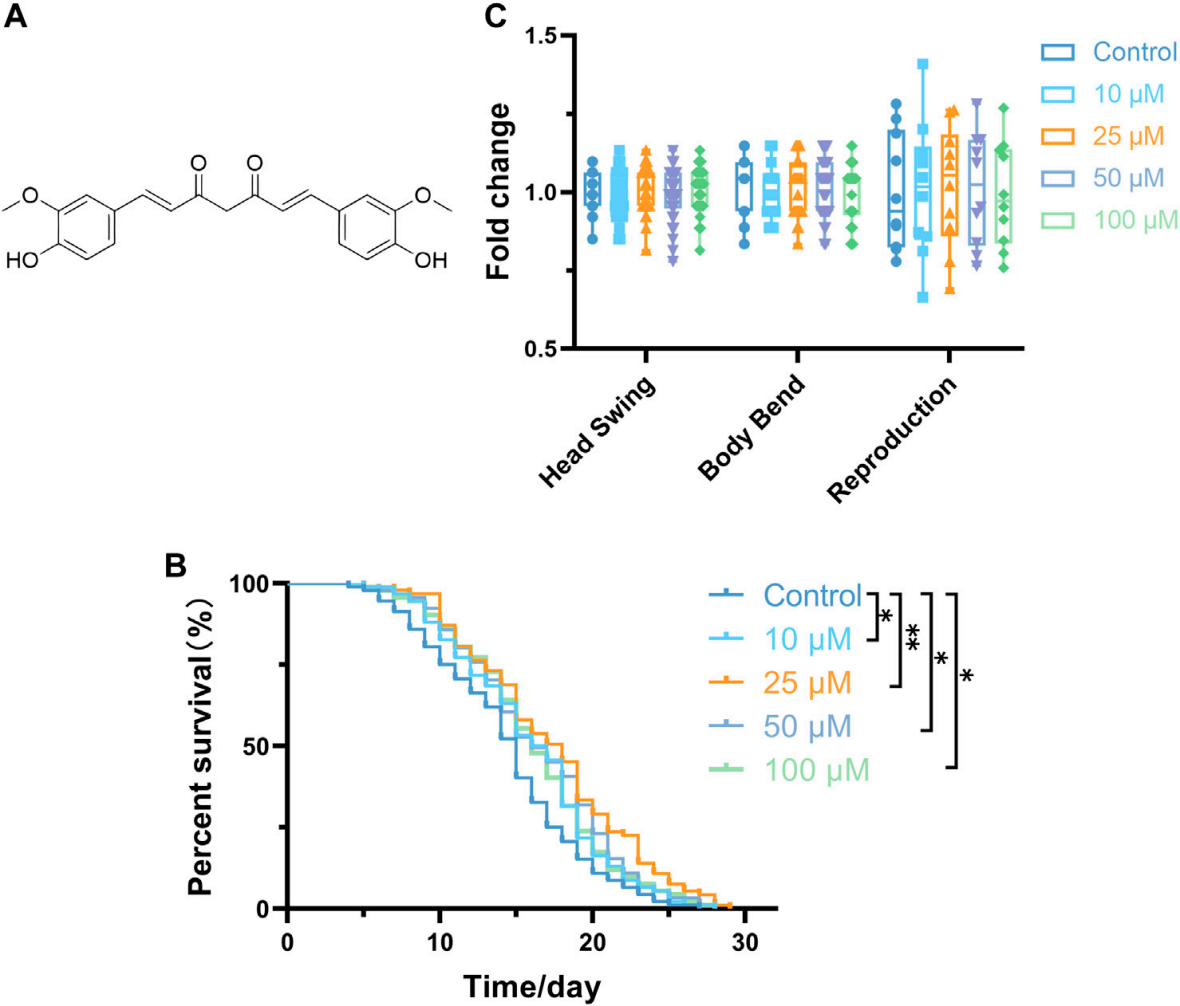
Curcumin increases *C. elegans* lifespan. **(A)** Chemical structural of curcumin. **(B)** Changes in the survival curves of wild-type L4 stage worms treated with 0.1% DMSO (carrier control) or with curcumin (10, 25, 50, and 100 µM) in N2 worms. **(C)** There was no effect of curcumin on nematode motility and reproductive capacity (*p* > 0.05) Statistical analysis was performed using GraphPad Prism software and *p*-values were calculated using a log-rank test. A *p* value < 0.05 was considered as a statistically significant difference.

The lifespan extension observed in the curcumin-treated groups was significantly different from the control group (0 µM). Notably, the most pronounced effect was observed at a concentration of 25 μM, which increased the average lifespan by 2.91 days (Figure 1B; Table 2). The survival curves demonstrated statistical significance based on a log-rank test. Notably, the proliferation of bacteria can affect the lifespan of *C. elegans* (Portal-Celhay, Bradley, and Blaser, 2012). To eliminate the effect of bacterial growth and metabolism on the lifespan of *C. elegans*, we explored whether curcumin affects the growth and metabolism of OP50. Results revealed that curcumin had no apparent inhibitory or growth-promoting effect on OP50 (Additional file Figure 1). These data confirm the capacity of curcumin to prolong the lifespan of *C. elegans*.

**TABLE 2:** Lifespan analysis: the effect of various concentrations of curcumin on the mean lifespan of *C. elegans*

Treatment	Mean	±SD	*p*-value (log-rank significance)
Control	14.435	4.9797	
10 µM	15.967	4.9513	0.0461
25 µM	17.344	5.5019	0.0001
50 µM	16.429	5.0949	0.0113
100 µM	16.163	4.9175	0.0322

#### 3.1.2 Curcumin did not affect the activity and egg-laying of *C. elegans*

The locomotor performance of *C. elegans* to some extent reflects their energy consumption (Yuan et al., 2012). Head swing and body bending frequency are indicators for evaluating locomotor behavior ability. The results showed that curcumin did not significantly change the head oscillation and body bending of nematodes, indicating that different concentrations of curcumin do not affect the locomotor ability of nematodes (*p* > 0.05) (Figure 1C). Here, the number of eggs laid in the experimental group remained unchanged compared to the control group. The number of eggs laid is usually used as an index to evaluate whether the drug is reproductively toxic to nematodes, and this experiment confirmed the safety of curcumin by detecting the number of eggs laid (Figure 1C). Based on the above results, curcumin prolongs the lifespan of nematodes and does not suppress nematode growth and development. Moreover, the effect of curcumin on nematodes is safe and harmless.

### 3.2 Curcumin improves the resistance of *C. elegans*

#### 3.2.1 Curcumin significantly reduces the ROS content in *C. elegans* under heat stress

**FIGURE 2:**
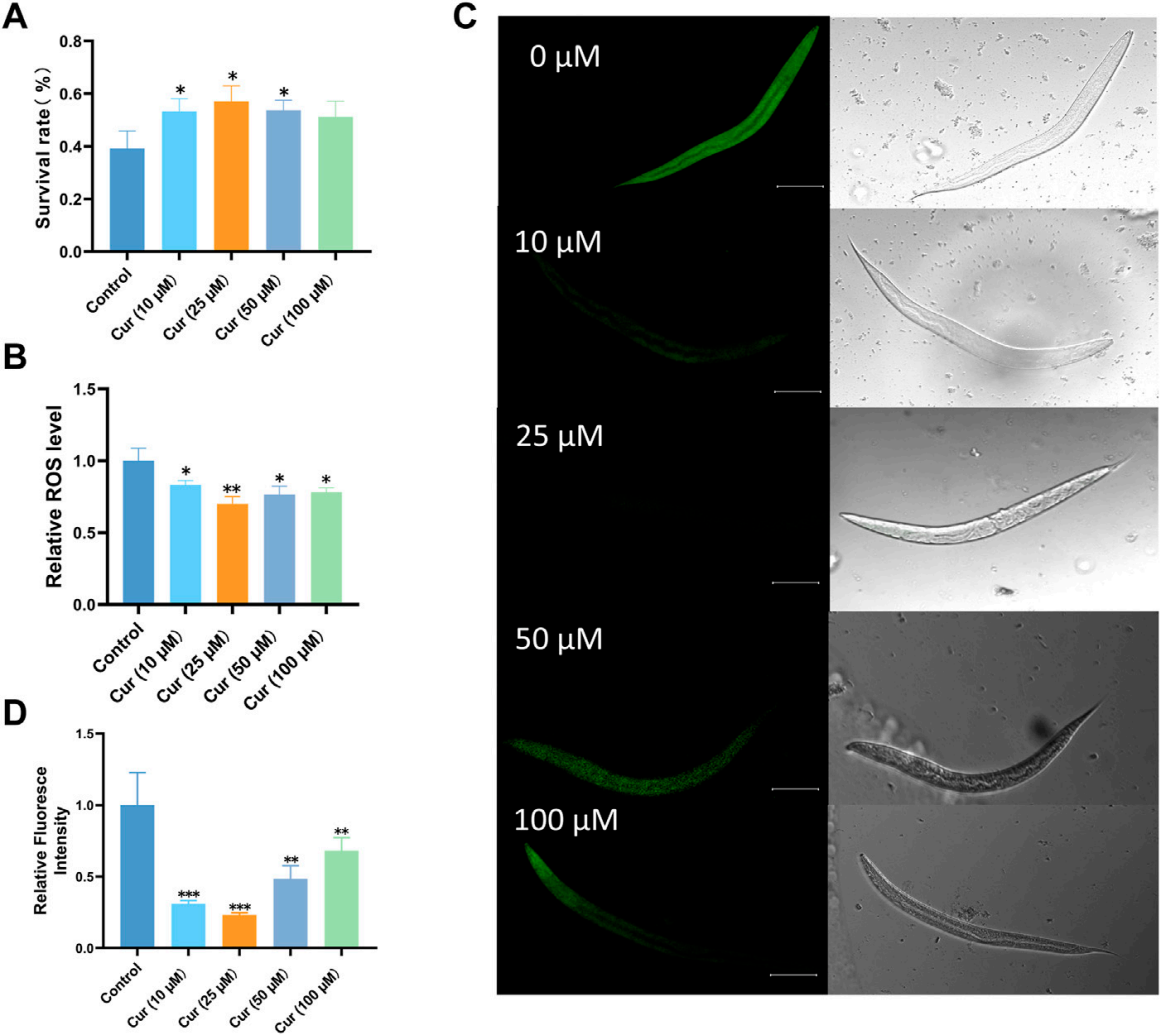
Curcumin enhances *C. elegans* resistance under heat stress (35°C) and oxidative stress. **(A)** Survival rate of heat-stressed *C. elegans* under different concentrations of curcumin intervention. **(B)** Different concentrations of curcumin reduced the intracellular ROS levels in *C. elegans* compared to the control group. The mean fluorescence intensity of each group (*n* = 30 *C. elegans*) is shown. **(C)** Representative fluorescence images of *C. elegans* under oxidative stress after treatment with different concentrations of curcumin. **(D)** ImageJ fluorescence quantification of *C. elegans*. Images were analyzed using ImageJ software, and numerical data were collected using GraphPad Prism software for statistical analysis. *p*-values were calculated using Student’s t-test. A *p* value < 0.05 was considered as statistically significant difference.

Increasing lifespan is associated with increased resistance to various forms of environmental stressors. For instance, heat stress causes mitochondrial damage through the accumulation of ROS (Slimen et al., 2014; Dilberger et al., 2019). Here, we treated L4 worms with heat stress (35°C) to investigate whether curcumin could enhance stress resistance. Interestingly, curcumin significantly increased the survival rate of *C. elegans* under heat stress. Survival of nematodes was significantly enhanced by curcumin at 10, 25, and 50 µM (*p* < 0.05) (Figure 2A). The highest survival rate of nematodes was observed at 25 μM, indicating that curcumin exerts protective roles against heat stress in *C. elegans*.

Moreover, the impact of curcumin on reactive oxygen species (ROS) levels was assessed using H2DCFDA. It was observed that curcumin at various concentrations effectively mitigated ROS levels in nematodes under heat stress conditions, with the optimal concentration being 25 µM. Consequently, curcumin treatment prominently attenuated the accumulation of ROS induced by heat stress (Figure 2B). These findings suggest that the enhanced longevity and stress resilience resulting from curcumin treatment can be attributed to its antioxidant properties (Pellavio et al., 2017).

#### 3.2.2 Curcumin significantly reduces the ROS content in *C. elegans* under paraquat stress

Paraquat promotes free radical generation. Under oxidative stress conditions, the number of free radicals in cells increases, causing structural and functional disruption of cell membranes, an increase in the content of lipid peroxides and a decrease in the content of antioxidant substances (Chinta et al., 2008). Paraquat-induced oxidative stress and significantly affects the lifespan and respiratory chain of nematodes (Dilberger et al., 2019). Therefore, we investigated whether curcumin treatment could reduce the level of ROS in N2 worms under oxidative stress. Confocal results revealed that different concentrations of curcumin were effective in reducing nematode ROS fluorescence (Figures 2C, D). Under paraquat-induced conditions, ROS levels in nematodes were significantly reduced in all four curcumin -treated groups compared with the control group. It showed that 25 µM curcumin significantly reduced the excess ROS accumulated in the body under oxidative stress, suggesting that curcumin may enhance the antioxidative stress effect of nematodes mainly by reducing ROS in the nematodes.

### 3.3 Curcumin enhances the T-AOC in *C. elegans*

**FIGURE 3:**
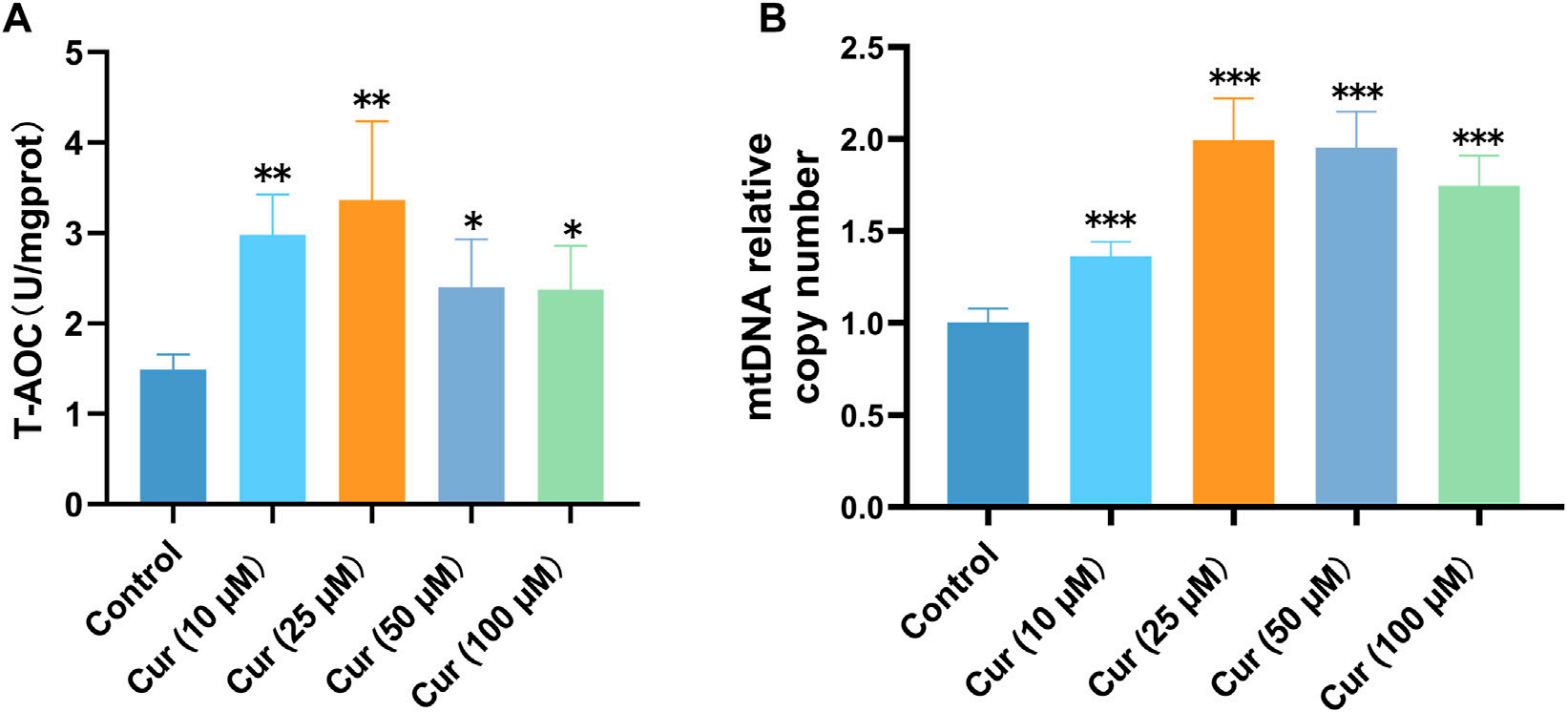
**(A)** Curcumin enhances the T-AOC in *C. elegans*. **(B)** Curcumin increased *C. elegans* mitochondrial copy number. The total DNA of *C. elegans* after pretreatment was extracted, and the levels of mtDNA and nDNA genes in *C. elegans* were detected by qRT-PCR. The changes in mtDNA relative to nDNA was calculated. Each set of experiments was repeated at least three times.

The antioxidant capacity of the body’s defense system is closely associated with the degree of health of the body. The body’s antioxidant system functions mainly through the elimination of free radicals, the decomposition of peroxides and the removal of catalytic metal ions, with various antioxidant enzymes, vitamins, amino acids and metalloproteins acting in synergy with each other (Yang et al., 2019). Compared with the control group, the T-AOC of *C. elegans* was significantly increased (10 µM *p* = 0.0056, 25 µM *p* = 0.0021, 50 µM *p* = 0.046, 100 µM *p* = 0.039), especially in the 10 and 25 µM curcumin-treated groups (Figure 3A). Therefore, we determined that curcumin enhanced the total antioxidant capacity of *C. elegans* by measuring the change in T-AOC activity.

### 3.4 Curcumin increases mitochondrial copy number in *C. elegans*

Given the significance of mitochondrial function in lifespan extension, this study aimed to investigate whether curcumin can enhance nematode lifespan in natural conditions by preserving mitochondrial integrity. Current studies suggest that mtDNA copy number variation in diseased tissues is closely associated with increased ROS production and the degree of aging and health status of individuals (Filograna et al., 2021). The pattern of high mtDNA copy number may be genetically related and is a favorable factor for familial longevity (Chiang et al., 2020). We discovered that different concentrations of curcumin could increase mitochondrial copy number (Figure 3B) in *C. elegans*, which was remarkable at 25 µM. This suggests that curcumin can increase the mtDNA copy number in nematodes, and by extension, the longevity of nematodes. Based on the above experimental results, curcumin at 25 µM concentration was considered optimal and was used as the intervention concentration in subsequent experiments.

### 3.5 RNA-seq results

#### 3.5.1 Identification of expressed transcripts

**FIGURE 4:**
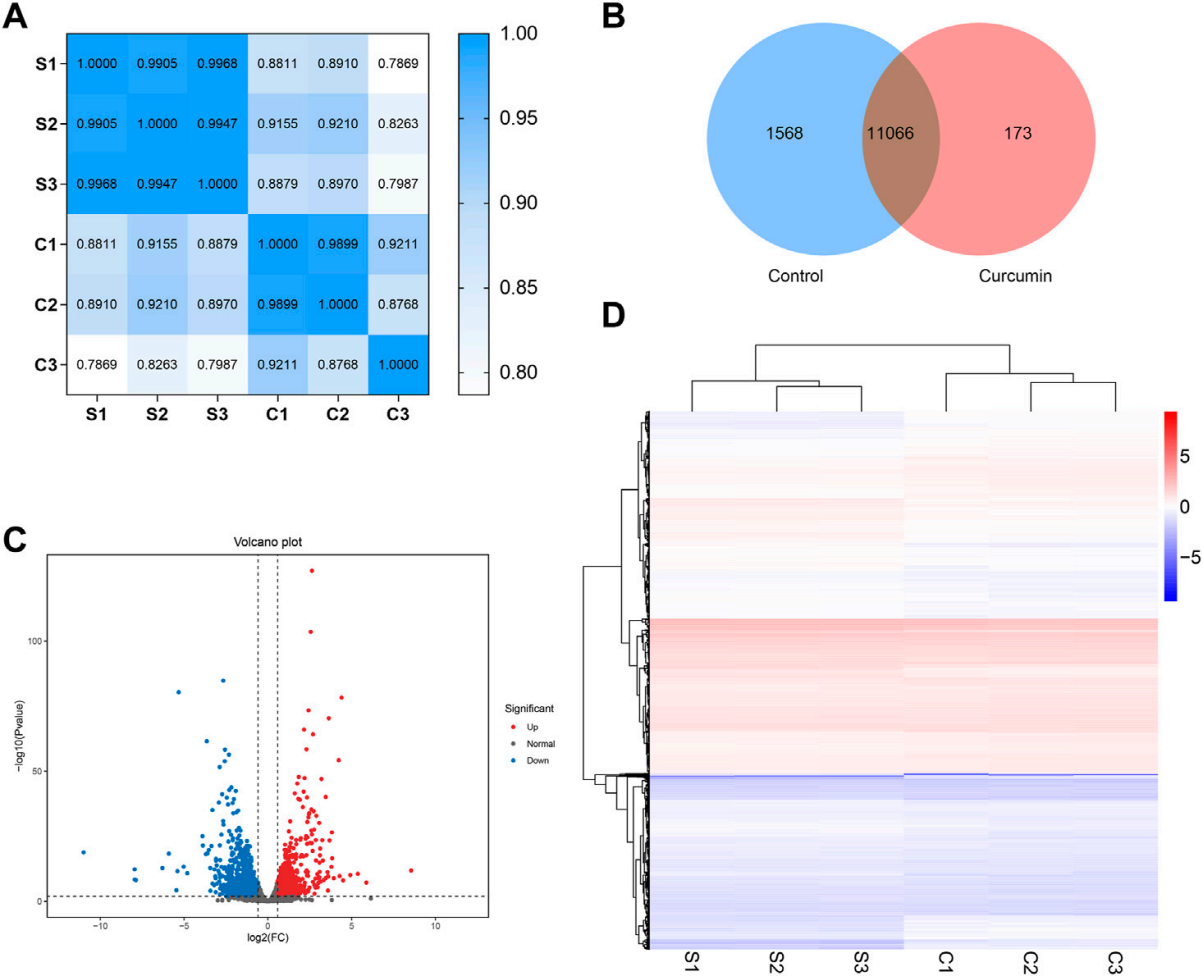
**(A)** Heat map of expression correlation between two samples. The values on each color block on the heat map in the figure represent the correlation between the two samples on the corresponding horizontal and vertical axes of the color block; the larger the value, the higher the correlation. The closer the Pearson correlation coefficient r obtained r2 is to 1, the stronger the correlation between the two replicate samples. **(B)** A Venn diagram showing differentially expressed genes. The differentially expressed genes of different comparative combinations are displayed in the Venn diagram, and the numbers on each region represent the number of genes under the corresponding classification, where the regions with overlap indicate the number of differential genes shared between related combinations in this region. **(C)** Differential expression volcano plot. Each point in the graph represents a gene, and the horizontal coordinate indicates the logarithm of the expression difference of a gene in two samples; the vertical coordinate indicates the negative logarithm of the statistical significance of the gene expression change. The blue dots represent downregulated differentially expressed genes, red dots represent upregulated differentially expressed genes, and black dots represent genes with no change. **(D)** Heat map of clustering of differentially expressed genes. The horizontal coordinates represent the sample names and the clustering results of the samples. The vertical coordinates represent the differential genes and the clustering results of the genes. The color represents the expression level of the gene in the sample log10 (FPKM+0.000001).

To investigate the potential life extension mechanism of curcumin, we extracted RNA from 2,000 *C. elegans* in each sample. The samples were named as follows: S1, S2, and S3 (all of which were not treated with curcumin) and C1, C2, and C3 (all of which were treated with curcumin of 25 µM). Since any biological experiment requires biological replication, the Pearson correlation coefficient (r) was used as an indicator for assessing biological replicate correlation, and the closer the calculated r2 was to 1, the stronger the correlation between the two replicate samples. The results showed that the RNA-seq data we obtained had good reproducibility and reliability (Figure 4A, Additional file: Supplementary Table S1).

#### 3.5.2 Differential gene identification

To investigate the effect of curcumin treatment on *C. elegans*, RNA-seq data were analyzed to establish the differentially expressed genes (DEGs) between treatment groups (Additional file: Supplementary Table S2). Figure 4B shows the Venn diagram of gene expression in curcumin-treated and untreated nematodes. Based on the criteria of fold change ≥2 and *p* ≤ 0.05, the distribution of DEGs in the curcumin-treated and control groups is illustrated in Figure 4C. Furthermore, cluster analysis was conducted to assess the expression patterns of DEGs in the samples. The clustering analysis depicted in Figure 4D demonstrates distinct expression patterns between the control and curcumin groups. These findings indicate that curcumin treatment influences gene expression patterns in *C. elegans*, thereby potentially modulating its biological functions.

#### 3.5.3 Functional distribution of DEGs

**FIGURE 5:**
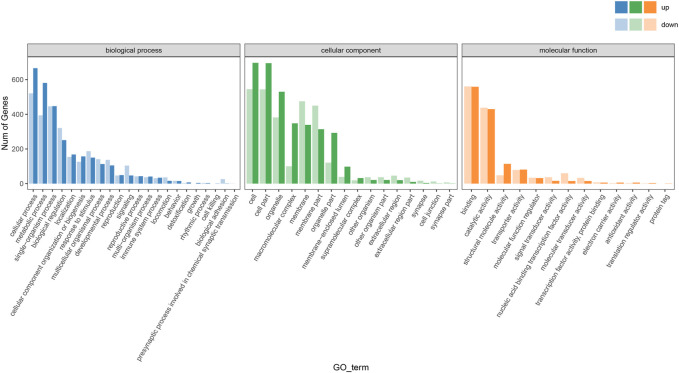
Statistical plot of GO secondary node annotation classification of differentially expressed genes in worms treated with curcumin (25 µM) and blank control. The horizontal coordinate is the GO classification, and the vertical coordinate is the number of differential genes.

**FIGURE 6:**
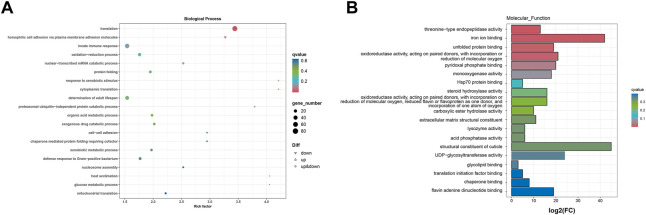
GO analysis of the genes significantly regulated in curcumin-treated *C. elegans*. **(A)** Differential gene biology process GO enrichment scatter plot. The horizontal coordinate is the ratio of the genes of interest annotated in the entry to the number of all differentially expressed genes, and the vertical coordinate is each GO annotation entry. The size of the dots represents the number of differentially expressed genes annotated in the pathway, and the color of the dots represents the q-value of the hypergeometric test. **(B)** Differential gene molecular function GO enrichment histogram. The horizontal coordinate is the number of genes of interest annotated in the entry, and the vertical coordinate is each GO annotation entry. The color of the bar represents the q-value of the hypergeometric test.

The enrichment of the GO pathway was analyzed based on the DEGs of curcumin-treated *C. elegans*, using threshold-corrected *p*-values < 0.05. In this work, 2516 GO items were significantly enriched based on DEGs (Additional file: Supplementary Table S3). The results showed that the biological processes primarily involved pathways including translation, homophilic cell adhesion via plasma membrane adhesion molecules, innate immune response, oxidation-reduction process, and protein folding (Figure 5, Additional file: Supplementary Figure S2). Cellular components primarily included ribosome, cytoplasm, proteasome core complex, cytosolic large ribosomal subunit, and small ribosomal subunit (Figure 6A, Additional file Supplementary Figure S3). Molecular functions mainly included oxidoreductase activity, acting on paired donors, with incorporation or reduction of molecular oxygen (GO:0016705), threonine-type endopeptidase activity (GO:0004298), unfolded protein binding (GO:0051082), iron ion binding (GO:0005506), and structural constituent of ribosome (GO:0003735) (Figure 6B; Additional file Supplementary Table S4). In the GO analysis, the molecular function was the most significant enrichment pathway, and all the pathways are closely linked to mitochondria. As the energy powerhouse of an organism, mitochondria supply energy to the organism through the tricarboxylic acid cycle and oxidative phosphorylation, where iron ions modulate the entire process of mitochondrial energy metabolism in bacteria. The unfolded protein reactions are the regulatory mechanisms of mitochondrial functional impairment (Tao et al., 2014; Pickles, Vigié, and Youle, 2018). The results of the analysis confirmed that curcumin may delay aging by regulating the energy metabolism of nematodes.

#### 3.5.4 Distribution of the KEGG-based pathway analysis of DEGs

**FIGURE 7:**
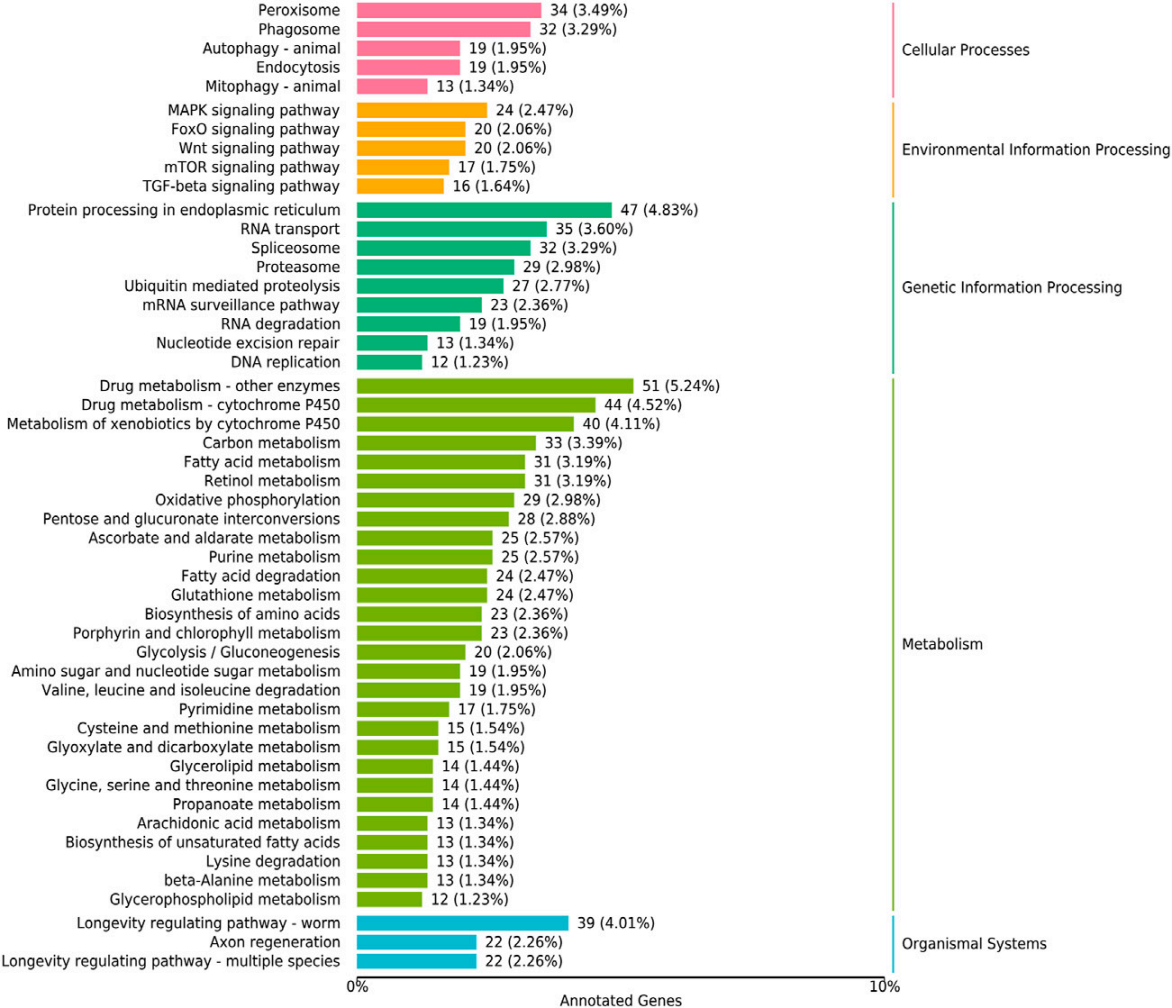
The KEGG analysis of differentially expressed genes in curcumin-treated *C. elegans*. The vertical coordinate is the name of the KEGG metabolic pathway, and the horizontal coordinate is the number of genes annotated to this pathway and its number as a percentage of the total number of genes annotated.

**FIGURE 8:**
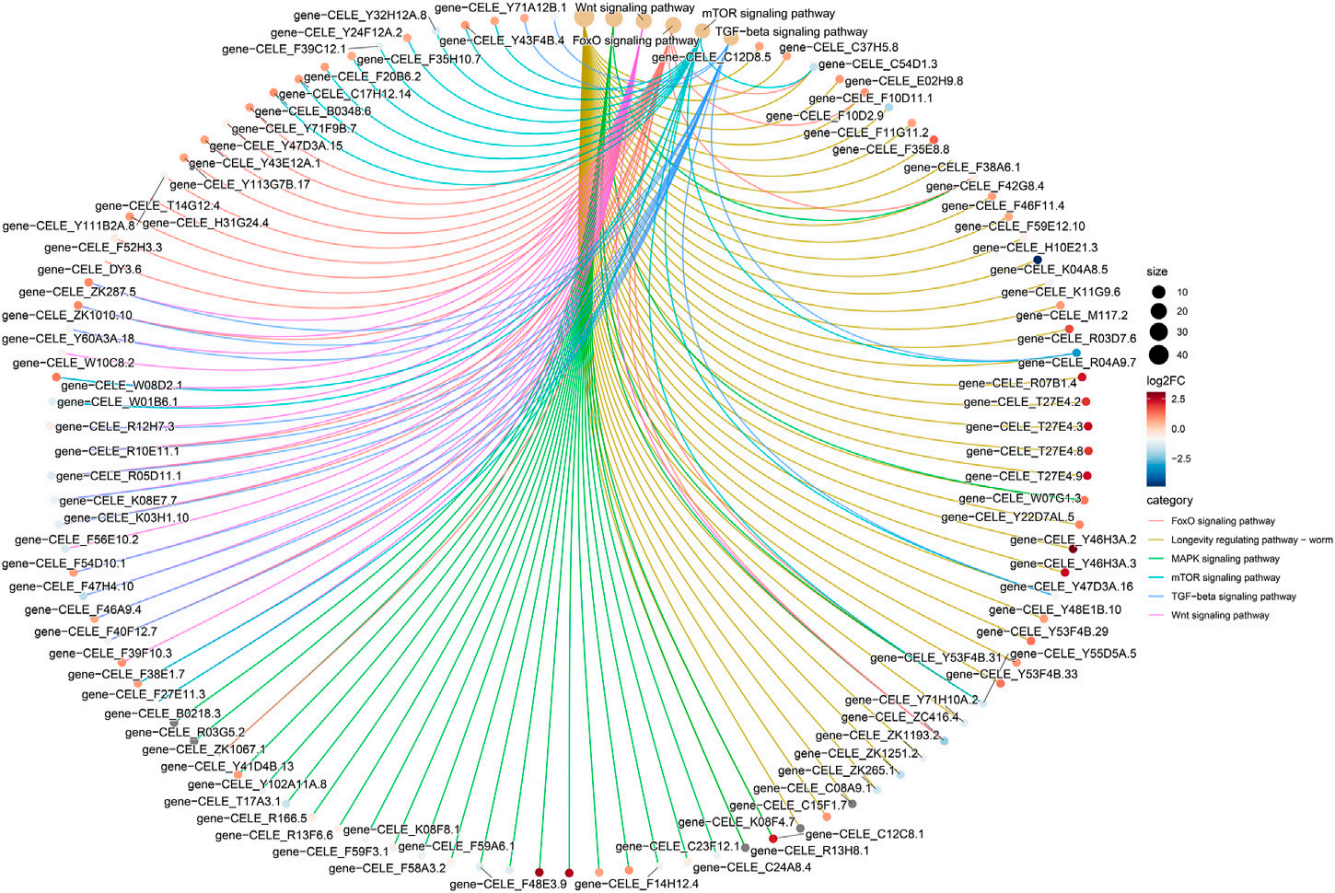
Differential gene KEGG enrichment network plot. The colors of the edges represent different pathways, and the colors of the gene nodes represent the differential ploidy, the larger the pathway node indicates the number of genes enriched to the pathway.

The KEGG-based pathway analysis using DEG data revealed that the nematode exposed to curcumin was significantly enriched in five pathways, i.e., MAPK, Forkhead box O transcription factors (FOXO), Wnt, mammalian target of rapamycin (mTOR), and transforming growth factor beta (TGF-β) (Figure 7; Additional file Supplementary Table S5). Figure 8 shows the association between genes interacting in the significant enrichment pathway and the longevity pathway. To further explore the mechanism of curcumin’s action in delaying aging, we further analyzed the relationship between the relevant signaling pathways and the nematode lifespan pathway (Additional file Supplementary Figure S4). We analyzed the expression of genes in the most enriched KEGG pathway and found that the expression associated with the MAPK signaling pathway was mostly downregulated. Moreover, the downregulation of MAPK-related genes was majorly concentrated in the p38 MAK kinase pathway (Additional file Supplementary Figure S5). The MAPK signaling pathway is essentially constituted of a three-tier kinase pattern that is extensively conserved in eukaryotes, including MAPK kinase kinase (MKK), MAPK kinase (MKK), and MAPK. These kinases are activated sequentially and simultaneously regulate a variety of important physiological and pathological processes in the body (Schinzel et al., 2019; Otarigho and Aballay, 2021). The present study discovered that curcumin can not only reduce ROS in nematodes but also inhibiting the MAPK signaling pathway, which can protect mitochondria from oxidative stress damage, and prolonging the lifespan of worms. The FOXO signaling pathway stimulates the expression of various functional genes related to DNA repair and free radical scavenging in the organism to induce apoptosis in abnormal or damaged cells. The oxidative stress-mediated balance of the FOXO/Wnt signaling axis plays an important role in maintaining the homeostasis of the intracellular environment (Bourgeois et al., 2021). Knockdown or mutation of different Wnt ligands in *C. elegans* can affect worm lifespan, indicating that changes in the Wnt microenvironment can also directly affect senescence (Li et al., 2022). mTOR is one of the major intracellular targets of IIS (insulin/IGF-1 signaling), which is triggered in response to adequate nutrient supply and growth factor signals. Mutant strains associated with mTOR have been found to alter nematode metabolism and extend lifespan (Nieto-Torres and Hansen, 2021). It has been found that there are multiple associations between TGF-β signaling and aging-related diseases. Abnormal TGF-β signaling can lead to cell degeneration, tissue fibrosis, decreased regenerative capacity, and abnormal metabolic function (Templeman et al., 2020).

### 3.6 Curcumin affects the expression of antioxidative stress-related genes and MAPK signaling pathway-related genes in *C. elegans*

**FIGURE 9:**
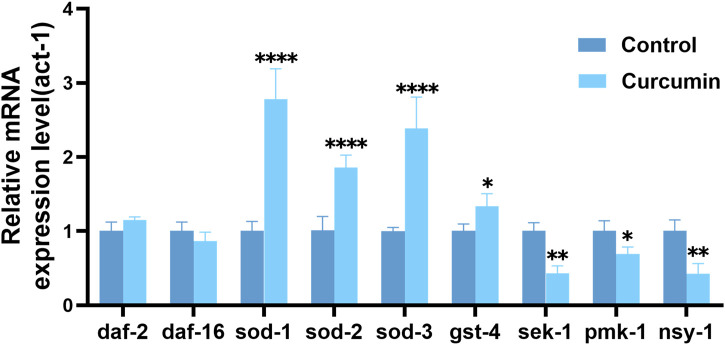
Curcumin can increase *C. elegans* longevity by regulating the expression of antioxidant-related genes and the MAPK signaling pathway related genes. Relative mRNA expression of daf-2, daf-16, sod-1, sod-2, sod-3, gst-4, pmk-1, sek-1 and nsy-1 genes in curcumin intervention (25 µM) and blank control (with act-1 as an internal reference gene) was determined.

Based on these results, we measured the expression of related genes. SOD-related genes are involved in the protection of *C. elegans* against oxidative stress. After curcumin treatment, the expression levels of genes sod-1, sod-2, sod-3, and gst-4 mRNA were significantly upregulated in the experimental group of worms compared to that in the control group (Figure 9). This indicated that the effect of curcumin on ROS may be due to the enhancing ROS clearance and regulation of GST-4 expression *in vivo* enhances oxidative stress resistance in *C. elegans*. SOD is the only known direct free radical scavenging enzyme that regulates the levels of superoxide and hydrogen peroxide generated by cells, which in turn regulates cell signaling (Brieger et al., 2012). GST is a class of multifunctional proteins that can protect the body from oxidative damage, mainly involved in the inhibition of oxidative stress (Urbizo-Reyes et al., 2022). Curcumin can prolong *C. elegans* lifespan by enhancing its antioxidant capacity.

The MAPK pathway is an important signaling system in eukaryotic cells, which includes a series of kinases with functions in regulating cell proliferation, differentiation, and apoptosis. ROS can activate the MAPK signaling pathway and affect various cellular activities (Liu et al., 2022). In *C. elegans*, the core MAPK signaling pathway comprises the nsy-1 gene encoding MKKK, the sek-1 gene encoding MKK, and the pmk-1 gene encoding MAPK (Li, Huang, and Liao, 2020). pmk-1 is critical as a pivotal gene for proper transmission of the p38 MAPK signaling pathway. It has been demonstrated that loss of pmk-1 gene function can inhibit copper and cadmium-induced apoptosis (Yarychkivska and Shaham, 2020). In contrast, the experimental findings provided further validation to the RNA-seq results by confirming the downregulation of nsy-1, sek-1, and pmk-1 gene expression in worms following curcumin intervention.

## 4 Discussion

### 4.1 *C. elegans* and aging

Aging is a complex process that is influenced not only by genetic factors but also by factors such as the environment and lifestyle. Organisms’ ability to regulate internal and external damage gradually weakens during their life cycle, leading to a physiological reduction of various biological activities (Hemagirri and Sasidharan, 2022). It is an inevitable and irreversible process of organism degeneration with age. At present, it is a hotspot to study the mechanism of senescence from the perspectives of heredity and environment. However, due to the complexity and diversity of the mechanism, the study of the mechanism is facing great challenges. As the first fully sequenced multicellular eukaryote, *C. elegans* has a simple structure, moderate life cycle, and strong reproductive capacity. Due to its decline in motor capacity, reduced feeding intake, and free radical accumulation, it has been widely used in aging and longevity research (Denzel, Lapierre, and Mack, 2019; Okoro et al., 2021). At present, the *C. elegans* model was widely used to screen and evaluate a variety of anti-aging compounds. Most of the studies on nematode aging are mainly focused on the genes regulating aging and the signal pathways regulating aging (Blackwell et al., 2019; Yuan et al., 2019; Hao et al., 2022).

Due to normal aging or adverse environmental stress, the body will produce excessive ROS, leading to the imbalance between ROS and antioxidant enzymes, causing oxidative stress, destroying protein, DNA, and other material structures, which will accelerate aging and apoptosis (Santos, Sinha, and Lindner, 2018). Therefore, to improve the antioxidant activity is an important way to delay aging and keep young and healthy. The antioxidative stress capacity and its lifespan are well correlated, so its oxidative stress capacity is often used as an indicator of a drug’s anti-aging capacity. Most of the anti-aging substances can mediate the overexpression of related proteins/enzymes in nematodes to eliminate ROS and improve the anti-oxidative damage ability. For example, the endogenous glutathione and antioxidant enzymes such as SOD, catalase (CAT), and glutathione peroxidase (GPx) play important roles in the detoxification of ROS. These enzymes can also reduce the damage caused by diseases such as diabetes, insulin cell damage, and atherosclerosis due to oxidative stress, and slow down the aging of the body (Wang et al., 2023). This study found curcumin can alleviate oxidative stress damage in *C. elegans*, enhance its antioxidant capacity and delay senescence.

### 4.2 Anti-aging effects of curcumin

Strategies that improve the antioxidant function of the body can be important avenues for preventing disease and prolonging life. As a kind of polyphenol, the curcumin is a natural product derived from long turmeric and is important for human health. Studies on curcumin anti-aging and geriatric diseases have shown that curcumin and its metabolites can extend the average life cycle of some animal models of aging. As a natural product, curcumin is rich in nutritional value with good antioxidant, antitumor, hypolipidemic, and immune-enhancing properties. Curcumin possesses a potent antioxidant effect, mainly through free radical scavenging to preserve cells from protein carbonylation, lipid peroxidation, and mitochondrial permeability shift (Pontes-Quero et al., 2021). In the studies on *drosophila*, yeast, and nematodes, curcumin treatment was found to extend lifespan in association with enhanced SOD enzyme activity (Zia et al., 2021; Utomo et al., 2022). By interacting with various antioxidant enzymes such as GPx and SOD, curcumin has the effect of reducing lipid peroxidation and preserving the active state of antioxidant enzymes (Wu et al., 2023).

In our study, four different concentrations of curcumin were used to intervene on nematodes. The results revealed that different concentrations of curcumin were able to significantly increase the longevity and resistance to aging of nematodes, with the best life extension effect obtained at an intervention concentration of 25 µM. This study demonstrates that curcumin significantly prolonged the lifespan of *C. elegans* by enhancing the antioxidant system of *C. elegans* to effectively resist free radical-induced damage in response to heat stress and oxidative stress. Meanwhile, after curcumin intervention, the increased expression of SOD-related genes and the enhancement of T-AOC content in *C. elegans* indicate that curcumin can significantly reduce the ROS content and enhance the activity of SOD and T-AOC (Figures 2, 3A). These results indicated that curcumin can enhance the oxidative stress ability of *C. elegans*. Curcumin also maintained mitochondrial function from being impaired and maintained the mitochondrial copy number at a relatively high level. Therefore, our current study provided evidence that curcumin can slow down the aging process by alleviating oxidation and enhancing the antioxidant capacity of the body.

Many age-related disorders are associated with mitochondrial dysfunction, measured by reduced energy production, structural damage to cellular components, and even cell death (Tyrrell et al., 2020). Mitochondrial oxidative stress damage is an important feature of aging and age-related diseases (Schmitt and Eckert, 2022). Moreover, the mtDNA copy number and its function decreased with age in short-lived medaka fish (Leuthner et al., 2021). We evaluated the mitochondrial copy number in *C. elegans* and observed that varying concentrations of curcumin led to a modest increase in mitochondrial copy number (Figure 3B). The primer sequences were designed carefully to measure the relative mitochondrial copy number relative to the nematode nDNA level. The mitochondrial copy number assay used in this experiment does not require extraction of nematode RNA. The degree of change in mitochondrial copy number is directly evaluated at the DNA level. Therefore, this method can reflect the mtDNA copy number, indicating that curcumin can influence the energy metabolism of mitochondria and maintain the mitochondrial copy number in *C. elegans* at a relatively high level. Several preclinical studies *in vitro* and *in vivo* have demonstrated the effectiveness of curcumin in various cancers. These effects may be related to the intricate crosstalk between curcumin-mediated mitochondrial turnover, autophagy, and apoptosis (Moustapha et al., 2015; Rainey, Moustapha, and Petit, 2020; Sala de Oyanguren et al., 2020).

### 4.3 Anti-aging molecular mechanism of curcumin

To further investigate the mechanism of action of curcumin, we performed the nematode transcriptome sequencing analysis. The results were analyzed using the GO and KEGG software. The GO enrichment analysis showed that the differences in gene expression were mainly in transcription-related biological processes, while the differences in cellular components were primarily enriched in the cytoplasm. The most significant enrichment pathway associated with molecular functions was linked to mitochondria (Figures 5, 6). The aging theory of “free radicals—mitochondria” holds that mitochondria are important sites of oxidative stress and oxygen free radicals, and mitochondria themselves are targets of free radical attack in the body (Olgar et al., 2020). Impaired mitochondrial function is a significant contributor to aging and age-related disorders. A decline in mitochondrial biogenesis is considered a prominent characteristic of the aging process (van de Ven, Santos, and Haigis, 2017).

The most representative pathway for environmental information processing was MAPK (Figure 7). The MAPK pathway is the oldest signal transduction cascade response in worms and has been identified as a key component of the immune response in *C. elegans* (Zhang S. Y. et al., 2023). The MAPK signaling pathway plays a vital role in regulating cell proliferation, differentiation, transformation, and apoptosis. This pathway is closely associated with the development of numerous diseases, including inflammation and tumors (Zhang X et al., 2023). Mounting evidence suggests that the dysregulation of MAPK is associated with biological processes leading to aging. The p38 MAPK is an important pathway of cellular senescence and is closely related to oxidative stress and inflammatory response. ROS are potential mediators of the MAPK signaling pathway (Abbas et al., 2022). ROS and phosphorylation of MAPK are involved in the induction of aging, leading to reduced protein and mRNA levels of acetyl coenzyme A carboxylase 1 (ACC1) (Marmisolle et al., 2017). The over-activation of MAPK enzymes and their lack of responsiveness may develop with aging and may result in promoting irreversible cellular senescence and accelerating the aging process (Wang et al., 2022). It is well known that p38 MAPK has an important role in accelerating cellular senescence. The upregulation of p38 MAPK contributes to premature aging. It is reported that the p38 MAPK inhibitor (SB203580) increased cell growth rate, and reduced stress cell morphology (Wang et al., 2021). Therefore, inhibition of the p38 MAPK signaling pathway may be a potential therapeutic approach to regulate aging and treat age-related diseases.

We performed qRT-PCR to further validate the mechanism of curcumin’s action. We examined the genes related to the SOD, GST and MAPK signal pathways in *C. elegans*. The results demonstrated that curcumin enhanced the resistance of *C. elegans* to oxidative stress. The expression of genes related to the MAPK signaling pathway activated by ROS was significantly downregulated. Oxidative stress plays a negative role in living organisms. In general, the cellular defense against ROS involves the removal of these harmful molecules through antioxidant mechanisms. During aging, ROS and other oxygen metabolites accumulate harmful proteins, lipids and DNA and weaken the antioxidant defense (Forni et al., 2019). In our current study, the expression of genes associated with MAPK-related signaling pathways was significantly downregulated after curcumin treatment. Therefore, it can be confirmed that curcumin prolonged nematode longevity via the MAPK signaling pathway.

## 5 Conclusion

This study demonstrated the efficacy of curcumin in mitigating heat stress-induced damage caused by free radicals through the enhancement of the antioxidant system in *C. elegans*. Curcumin significantly reduced the ROS content and enhanced the activity of SOD in *C. elegans*, enhancing their resistance to oxidative stress and significantly prolonging *C. elegans* lifespan. Curcumin also enhanced the antioxidant capacity and maintained the mitochondrial copy number at a relatively high level. The RNA-seq and qRT-PCR results confirmed that the life-prolonging effect of curcumin was mainly related to the MAPK signaling pathway. These are important factors that enable curcumin to positively affect *C. elegans* longevity. The present study also found that curcumin affects the functional state of *C. elegans* related to mitochondrial function, while the specific mechanism of its influence on mitochondrial function and the specific intracellular pathways of action will be a key question for further exploration and study in the future.

## Data Availability

The datasets presented in this study can be found in online repositories. The names of the repository/repositories and accession number(s) can be found below: NCBI BioProject [https://www.ncbi.nlm.nih.gov/bioproject/], PRJNA949877.
