# Supplementary material for: Curcumin supplementation increases longevity and antioxidant capacity in Caenorhabditis elegans
Source: Front Pharmacol. 2023 Jun 6;14:1195490. doi: 10.3389/fphar.2023.1195490 (PMC10279890; doi:10.3389/fphar.2023.1195490)
Supplement: Supplementary file 1 [file DataSheet1.zip › Additional file/1-Supplementary figure.docx]

Supplementary Material

Curcumin Supplementation Increases Longevity and Antioxidant Capacity in *Caenorhabditis elegans*

Jianing Xu ^1^, Pengyun Du ^1^ ，Xiaoyu Liu ^1^ ，Xiao Xu^1^ ， Yuting Ge ^1^， Chenggang Zhang ^1*^

^1^ School of Life Sciences, Beijing University of Chinese Medicine, Beijing 102488, China

*** Correspondence:**

Chenggang Zhang

jxs@bucm.edu.cn

# 1 Supplementary Figures and Tables

## 1.1 Supplementary Figure 1

**Curcumin has no inhibitory effect on the growth of OP50**

**Supplementary Figure 1:** To study whether curcumin has an inhibitory effect on the growth of OP50, we conducted an inhibition ring experiment, and the experimental results showed that the curcumin group, water group and fungicidin group all had no effect on the growth of OP50. There was no appearance of an inhibition ring on the LB medium. Images were analyzed using ImageJ software, and numerical data were collected using GraphPad Prism7 software for statistical analysis. The experiment was repeated seven times.

## 1.2 Supplementary Figure 2

**Statistical plots of COG annotation classification of differentially expressed genes**

**Note:** The abscissa is the classification of COG, and the ordinate is the number of genes.

## 1.3 Supplementary Figure 3

**GO enrichment network plot of the differential gene on cell components**

**Note:** The abscissa is the classification of COG, and the ordinate is the number of genes.

## 1.4 Supplementary Figure 4

**KEGG pathway map of the MAPK and FOXO signaling pathway of differentially regulated genes.**

**Note:** Green indicates downregulated genes, and red indicates upregulated genes.

## 1.5 The flowchart of curcumin supplementation increases longevity and antioxidant capacity in caenorhabditis elegans.

## 1.6 Supplementary Table 1

**The sequencing data quality assessment statistics for all the samples**

| SampleID | ReadSum | BaseSum | GC (%) | Q20 (%) | Q30 (%) |
| --- | --- | --- | --- | --- | --- |
| C1 | 20241811 | 6054553612 | 46.28 | 96.63 | 91.07 |
| C2 | 25446173 | 7613447202 | 46.23 | 96.38 | 90.47 |
| C3 | 27536654 | 8228807822 | 46.4 | 96.84 | 91.46 |
| S1 | 20804992 | 6218400336 | 45.84 | 98.52 | 95.23 |
| S2 | 20548036 | 6149819782 | 45.74 | 98.56 | 95.43 |
| S3 | 20974837 | 6272212682 | 46 | 98.38 | 94.94 |

**Note:** SampleID：Sample analysis number; ReadSum：Total number of pair-end Reads in the Clean Data; BaseSum：Total base number in Clean Data; GC (%); GC content in Clean Data, where the two bases of G and C account for the total base in Clean Data percentage; Q20(%) Clean Data：Percentage of bases with mass values greater than or equal to 20; Q30 (%) Clean Data：Percentage of bases with mass values greater than or equal to 30

## 1.7 Supplementary Table 2

## Results of differentially expressed genes

## Note: The first column shows the gene ID, and the next columns show the counts and expressions of the genes in each group and the differential expression information, including FDR, Fold Change (FC), and regulatory information (up: upregulated expression, down: downregulated expression).

## Note: log2FC is not directly calculated by FPKM value, but the read count value is converted by DESeq2 or EBSeq software, and the converted count value is used to calculate the log2FC. This calculation method is more accurate, but there is a small difference with FPKM value.

## See Additional file 2- DEG file for details.

## 1.8 Supplementary Table 3

## Statistics of GO secondary node annotation of differentially expressed genes

**Note:** The first column indicates the classification of GO primary nodes (cellular component, molecular function, biological process). The second column indicates the classification of secondary nodes under GO primary nodes. The third column indicates the number of genes annotated to the corresponding GO secondary nodes. The fourth column indicates the number of differentially expressed genes annotated to the corresponding GO secondary nodes. The fifth column indicates the ID of differentially expressed genes annotated to the corresponding GO secondary nodes, and #Total_gene indicates the total number of genes.

**See additional file 3-GO file for details.**

## 1.9 Supplementary Table 4

**GO enrichment analysis of differentially expressed genes in Fig. 5C**

**Note:** **ID:** GO term's ID. Description: Functional description of the GO term. Gene Ratio: Number of differential genes enriched in the term of the GO/Number of differential genes enriched in the corresponding primary category. Bg Ratio: Total number of genes with this function/number of all genes. Enrich factor: enrichment factor. P value: The enriched P value. Q value: q value. Gene ID: The specific gene id annotated to the term.

**See additional file 4-GO Molecular file for details.**

## 1.10 Supplementary Table 5

**KEGG pathway annotation results statistics file**

**Note:** # KEGG_n Number: Differentially expressed genes annotated in all pathways; KEGG_N: Number of genes annotated in all pathways; #Pathway: KEGG pathway name; ko_ID: KEGG Orthology, i.e. KEGG direct homolog ID; DE gene: Number of all differentially expressed gene(s) annotated to the pathway; gene: Number of genes annotated to the pathway; DEgene_all; Number of differentially expressed genes annotated to all KEGG pathways; gene_all: Number of genes annotated to all KEGG pathways. Gene_id names are annotated to the differentially expressed genes of the pathway. KOs are annotated to the K number corresponding to the differentially expressed gene of the pathway

## See Additional file 5-KEGG file for details.

## 1.11 Supplementary Table 6

**Gene ID information on the relationship between environmental information processing pathways and aging pathway**

## See Additional file 6-Gene ID file for details.

## 1.12 Certificate of curcumin assay
